# Psychological insight into corruption: construction and validation of the Corrupt Intention Scale (CIS)

**DOI:** 10.1186/s41155-025-00352-3

**Published:** 2025-05-20

**Authors:** Carlos Ramón Ponce-Díaz, Jesús Joel Aiquipa-Tello, Edgard Fernando Pacheco-Luza

**Affiliations:** Instituto Científico de Investigación, Universidad Andina del Cusco, Urb. Ingeniería Larapa Grande A-7, San Jerónimo, Cusco, Peru

**Keywords:** Corrupt behavior, Corrupt intent, Theory of Planned Behavior, Assessment, Psychometric properties

## Abstract

**Background:**

Corruption, as a psychosocial problem, impacts institutional stability and interpersonal trust. From a psychological perspective, the study of this phenomenon has focused on analyzing corrupt behavior. However, empirical evidence on the measurement of corrupt behavior remains limited due to its covert nature. An alternative strategy is to assess corrupt intent, as it allows inferring behavioral patterns without resorting to direct observation of these acts.

**Objective:**

The aim of this study was to construct and validate the Corrupt Intention Scale (CIS), taking the theory of planned behavior as a framework.

**Methods:**

We conducted a multiple study with a total of 1, 488 Peruvian adults. Multivariate statistical techniques such as exploratory and confirmatory factor analysis were used.

**Results:**

We found that the CIS presented evidence of internal structure validity for a 12-item model grouped into three correlated factors. It showed measurement invariance with respect to sex, convergent validity and satisfactory reliability.

**Conclusion:**

The CIS presents psychometric properties that support its use to measure corrupt intent, and can be used in evaluation, research and prevention contexts. In a global context where corruption remains a critical challenge, reliable and valid measures strengthen the basis for future research and mitigation programs.

**Supplementary Information:**

The online version contains supplementary material available at 10.1186/s41155-025-00352-3.

## Introduction

According to Transparency International (TI, 2009), corruption is the abuse of entrusted power for private gain. Corruption can occur anywhere (e.g., companies, government, and civil society), can involve anyone (e.g., politicians, officials, and the public), and can include behaviors such as public workers demanding or receiving money or favors in exchange for services, officials granting jobs or contracts to family or friends, and companies or individuals bribing officials to obtain lucrative agreements or advantages in accessing services (TI, 2009).

The ethical, social, political, and economic implications of corruption have led the scientific community to become interested in studying it and to put forth explanations for it. The study of corruption requires an interdisciplinary approach. Disciplines such as political science, sociology, history, anthropology, economics, and psychology analyze the phenomenon to determine its ontological bases, processes, and intrinsic dynamics, as well as its impact on society.

A psychological approach to corruption is fundamental, since a corrupt act is a human behavior and as such has analyzable psychological components. In this regard, corrupt behavior is a particular type of dishonest or unethical conduct, as it involves a transgression of social norms and rules. However, unlike other dishonest behaviors (e.g., deception or lying), a corrupt act involves the abuse of power and is considered more severe due to the consequences it has at the individual, group, and societal levels (Modesto & Pilati, 2020). Therefore, psychological research on this type of behavior, together with the study of the associated individual, interpersonal and group psychological variables, is essential both to understand its nature and to implement anti-corruption policies.

Individual psychological variables, such as narcissism, psychopathy and machiavellianism (Gu et al., 2021; Nugraha & Etikariena, 2021; Zhao et al., 2016), dispositional greed (Li et al., 2023), and beliefs in a just world (Modesto et al., 2020), represent relatively stable patterns of thinking and behavior that may increase the likelihood of corrupt acts. It has also been reported that power can foster corruption, either mediated by individual traits (Hu et al., 2024) or by cultural factors (Cai et. al., 2024). Interpersonal variables, such as personal control and reciprocity (Su et al., 2023) and social value orientation (De Waele et al., 2021), may justify and encourage participation in corrupt behavior in pursuit of personal benefits. In turn, group variables, such as descriptive norms (Zhao et al., 2019), ethical and organizational culture (Resende et al., 2024; Zahari et al., 2022), and meritocratic ideologies (Tan et al., 2017), may influence corrupt behavior, as individuals tend to adapt their behavior to group norms, even when these involve immoral or illegal actions.

To optimize the investigation of corrupt behavior, it is necessary to have rigorous instruments for this purpose. Thus, several tools have been developed in the scientific literature to measure corrupt behavior and related aspects. These include the Intercultural Business Corruptibility Scale (IBCS; Leong & Lin, 2009), the Corruption Awareness Scale (CAS; Tan et al., 2016), the Corruption Propensity Scale (CPS; Agbo & Iwundu, 2016), the Defining Issues Test (DIT-SF; Guerrero-Martelo et al., 2018), and the Attitudes Toward Corruption Scale (SATC; Orellana & Bossio, 2021).

Regarding the referenced tests, only the CPS and the DIT-SF present the theoretical foundations of the measured construct, namely moral development and the trait-based or propensity approach, respectively. Additionally, some methodological limitations were identified, such as the use of non-representative samples, specifically university students, in the CPS and DIT-SF. The absence of procedures to evaluate validity based on internal structure in the CAS was also noted. The procedures for factor rotation or extraction were not reported when applying exploratory factor analysis (EFA) in the IBCS and DIT-SF, and confirmatory factor analysis (CFA) was not used in the CAS and DIT-SF. Furthermore, CFA analyses were reported, although the procedures underlying these analyses corresponded to EFA in the IBCS and SATC. Finally, all instruments reported only the alpha coefficient as an indicator of reliability, an estimator that is insufficient for evaluating this property, as the necessary requirements, such as unidimensionality and tau equivalence, are difficult to meet in practice.

Recently, a study examined several instruments developed or adapted to assess corrupt behavior, including the aforementioned tests (Ponce-Díaz et al., 2024). Among the most relevant findings, it was identified that many of these instruments lacked an explicit theoretical framework in their design and that their psychometric properties were incompletely reported. These results suggest that, at present, there is no consolidated test to measure corrupt behavior, which could be attributed to the complexity of identifying and operationalizing observable indicators that reflect the presence of such behavior. In this context, a possible alternative is to assess corrupt behavior through the intention to carry it out.

According to the Theory of Planned Behavior (TPB; Ajzen, 1991), intention precedes the execution of actual behaviors: the stronger the intention, the greater the likelihood that the behavior will be performed. In this framework, intention is influenced by three key factors. The first factor is the attitude toward the behavior (ATB), defined as the degree to which performing an action is evaluated positively or negatively. The second factor is the subjective norm (SN), which refers to the perceived social pressure to engage in or refrain from a particular behavior. The third factor, perceived behavioral control (PBC), refers to an individual’s perception of their ability to carry out a specific behavior. Each of these factors is determined by a set of beliefs, including behavioral beliefs, normative beliefs, and control beliefs. In the case of PBC, it is important to highlight that it has been used as an alternative indicator of actual behavioral control (Ajzen, 2020), given that measuring actual control can be complex or even unfeasible. For this reason, most studies employ PBC as a representative measure of actual control in research on human behavior (Ajzen, 2020; Hagger et al., 2022).

Among the reviewed models explaining general and dishonest behavior—such as Festinger’s (1957) cognitive dissonance theory, Tajfel and Turner’s (1979) social identity theory, Bandura’s (1999) moral disengagement theory, and Ashton and Lee’s (2008) trait perspective—we consider the TPB to be the most appropriate framework for our purposes. This choice is based on several reasons: first, it provides a robust psychological approach to behavior analysis through the construct of intention; second, it integrates attitudinal, cognitive, and normative components coherently; third, it is used in a variety of contexts, such as adolescent safe sexual behavior (Rumenge et al., 2025), road safety (El Hafidy et al., 2024), and pedagogical innovation (Tang et al., 2025); and finally, it is backed by strong empirical evidence, as demonstrated by numerous synthesis studies (e.g., Hagger et al., 2022; Lidong et al., 2025; Somoray et al., 2024).

In congruence with the TPB, corrupt intent refers to a person’s propensity, willingness or commitment to use his or her position or power (of any kind) to obtain a perceived personal or intragroup benefit (Vilanova et al., 2022). Therefore, the TPB approach allows quantifying a person’s willingness to engage in corrupt behavior under certain conditions.

The objective of this study was to construct and validate the Corrupt Intention Scale (CIS). In Study 1, based on the discussed theory, we designed a scale to measure corrupt intent and determined its factorial structure and reliability. In Study 2, we evaluated the factorial structure of the CIS obtained in Study 1, and we tested alternative models suggested by confirmatory factor analysis. In Study 3, we determined the measurement invariance for sex and obtained evidence of its validity based on its relationship with other variables (Machiavellianism, narcissism, psychopathy, dispositional greed, and dispositional envy).

We believe that the construction and validation of a corrupt intent scale within the framework of Goal 16 of the Sustainable Development Goals (SDGs), focused on promoting just, peaceful and inclusive societies, is essential to address behaviors that hinder its fulfillment. Corruption, in its various forms, compromises resources, equity and institutional trust, hindering sustainable policies. A validated instrument will identify patterns and underlying factors, facilitating the design of preventive and corrective strategies that promote more transparent and accountable systems, aligned with the principles of the SDGs.

## Study 1

### Methods

#### Participants

A total of 369 adults (60.7% women, 39.3% men) aged 18 to 77 years (*M*_age_ = 29.5, *SD*_age_ = 15.6) participated (see Supplementary Material 1). A total of 33.9% reported residing in Lima, and 66.1% reported residing in other departments of Peru. The majority of participants reported a higher level of university instruction (77.8%). A total of 69.1% were university students, 18.7% were dependent workers, 6.2% were independent workers, and 10.4% had no occupation. Most of the participants identified themselves as mestizo (51.8%), and only 3.5% belonged to a political party. The sampling was nonprobabilistic.

The sample size was determined based on methodological guidelines derived from simulation studies (MacCallum et al., 1999; Mundfrom et al., 2005), aiming to minimize the impact of sample size on the quality of the exploratory factor analysis (EFA) solutions. A subject-to-item ratio of 7:1 was selected, which falls within the recommended range of 5:1 to 10:1. Given a 47-item scale, the minimum required sample size was 329 participants, a threshold that was exceeded in the current study. Additionally, we ensured overdetermination of the common factors by including at least seven variables per factor, surpassing this criterion by including eight items in the factor with the fewest variables. The level of item communality was also considered, with the assumption that over 50% of the items had communalities greater than 0.40. Lastly, we assessed the Kaiser–Meyer–Olkin (KMO) index and Bartlett’s test of sphericity to confirm the adequacy and sufficiency of the sample size for conducting the EFA.

#### Instruments

Sociodemographic data form. A form was created to collect data on sex, age, place of residence, education level, occupation, perceived ethnic self-identification, and political group affiliation.

Corrupt Intention Scale (first version). The CIS had 47 items that were theoretically grouped into three factors to measure corrupt intent. The scale was constructed based on TPB (Ajzen, 1991). The type of response was polytomous, with a 5-point Likert scale (1 = *totally disagree to* 5 = *totally agree*).

#### Procedure

Our research was not based on personally identifiable data that constitute an exception to ethical approval according to the Colegio de Psicólogos del Perú (2018). Therefore, ethical approval was not required. Despite this, for the entire study, we considered the ethical scopes of the Code of Ethics of the World Medical Association (World Medical Association, 2013) and the Colegio de Psicólogos del Perú (2018). Written informed consent to participate in the study was obtained from study participants, who were guaranteed that they would not be harmed, that they would stay anonymous, that their data would be confidential, and that their data would be used for research only.

The development of the CIS was conducted in several stages, adhering to the guidelines proposed by the American Educational Research Association et al., (2014) and Muñiz and Fonseca-Pedrero (2019). In the first phase, a review of empirical studies on corrupt behavior from the past five years, in both English and Spanish, was undertaken. The aim of this review was to identify the instruments used to measure corrupt behavior, as well as the underlying theories and their psychometric properties. The studies reviewed revealed that corrupt behavior has been assessed through questionnaires, corruption scenarios, and corruption games. Additionally, the tests evaluated constructs such as corrupt acts, corruption perception, attitudes toward corruption, corruption propensity, and corrupt intention. However, no single test was found that comprehensively assessed corrupt behavior. Among the limitations identified in many of the tests reviewed were the lack of explicit theoretical support in the construction of the instruments, the partial reporting of the methodological processes involved in developing the tests, or the complete absence of such information. For more detailed information on this review, refer to Ponce-Díaz et al., (2024).

The results of the review supported the creation of the CIS and provided a foundation for considering key aspects in its development process. These aspects include: a) selecting a theoretical framework that supports the construct being measured; b) designing the items to assess indicators of the chosen construct while avoiding the explicit use of terms related to corruption or corrupt behaviors; c) systematically incorporating as much validity evidence as possible; and d) applying a variety of statistical methods appropriate to the nature of the data.

In the second phase of instrument development, the TPB (Ajzen, 1991) was selected and adapted as the conceptual framework for measuring corrupt intent. This theory provided the foundation for structuring the questionnaire. Accordingly, we designed 47 items distributed across three key theoretical dimensions: 25 items to assess perceived behavioral control (PBC), 14 items to measure attitude toward the behavior (ATB), and 8 items to capture subjective norms (SN). The items were designed to accurately reflect the theoretical constructs of the model while avoiding terms such as “corruption,” “corrupt,” “corrupt act,” or “corrupt behavior,” as well as any similar expressions that could introduce response bias due to social desirability.

We ensured that the selection of the number of items for each factor of the TPB (Ajzen, 1991) responded to its conceptual complexity and expected variability in measurement. PBC required 25 items due to its multidimensional nature, encompassing perceived ease/difficulty of behavior and access to resources and skills. In contrast, the ATB dimension was assessed with 14 items, since it is less complex than perceived behavioral control. ATB reflect personal judgments and emotional reactions to behavior, which allows them to be measured effectively with a moderate number of questions. Finally, SN was measured with eight items, since they represent a more specific and homogeneous construct, justifying a smaller number of questions.

In the third phase, we applied the first version of the CIS to the first sample. We use Google Forms to present the instructions, items, and response options online. The link was shared via social media and email. The application database was downloaded into Excel, totaling 409 responses. Forty responses were discarded because the respondents were under 18 years of age. There were no missing answers given the configuration we used in Google Forms*.* Thus, a database with 369 cases was obtained.

The fourth and fifth phases are described in Studies 2 and 3, respectively.

#### Data analysis

Descriptive statistics are reported for the variables. The Anderson‒Darling test and Mardia coefficient were used to evaluate whether the univariate and multivariate distributions were close to normal, respectively.

To obtain internal structure validity indicators, the homogeneity index or item-test correlation coefficient (*r*_it_) was calculated, adequate values being defined as those greater than 0.30, as well as EFA, following the scope of Lloret-Segura et al. (2014). To verify the goodness of fit, the KMO index and the Bartlett’s test of sphericity was determined. The method used in the EFA was the minimum residuals method, with direct oblimin rotation. The number of factors to be extracted was determined through the parallel analysis criterion.

Reliability was determined using the internal consistency method, specifically through the *ω* coefficient (McDonald, 1999) with its 95% confidence interval. To calculate the estimators in this and the other two studies, the R program version 4.1.2 (R Development Core Team, 2021) was used, specifically the packages MVN (Korkmaz et al., 2014), lavaan (Rosseel, 2012), and semTools (Jorgensen et al., 2022) (see Supplementary Material 2).

### Results

The highest response mean was that of Item 26 (*“For me, keeping myself free of corrupt acts is extremely valuable”*; *M* = 4.13; *SD* = 1.08), while the lowest was for Item 8 *(“I believe that unlike other problems, corruption is not serious”*; *M* = 1.76; *SD* = 1.13). The asymmetry values varied between -1.27 (Item 26) and 1.47 (Item 8), while the kurtosis values ranged between -1.17 (Item 31, *“To receive a better public service, you have to give something in return”*) and 1.23 (Item 8). Both the Anderson‒Darling test (values between 44.7 and 11.9, *p* < 0.001) and the Mardia coefficients for skewness (29322.1, *p* < 0.001) and kurtosis (48.9, *p* < 0.001) indicated a different distribution from the univariate normal and multivariate. Likewise, 14 items had *r*_*it*_ values lower than 0.30, so we decided to eliminate them (items 2, 8, 9, 15, 16, 17, 18, 19, 20, 25, 26, 27, 28 and 30). After this process, the second version of the CIS had 33 items.

This second version was submitted to EFA. Correlation coefficients between items ranged from 0.07 to 0.80 (see Table A of Supplementary Material 3). The communalities ranged from 0.21 to 0.81, with nine items had communalities between 0.21 and 0.40, 10 items between 0.41 and 0.59, and 14 items between 0.60 and 0.81. The goodness-of-fit measures were adequate (*χ*^*2*^ = 6258.5, *df* = 528, *p* < 0.001; *KMO* = 0.94).

The initial EFA suggested a four-factor solution. However, items with cross-loadings, factor loadings below 0.40, and imprecise or ambiguous content were removed to enhance the clarity and precision of the instrument. This process was conducted iteratively, refining the model at each stage to optimize the factorial structure and ensure that the retained items accurately represented the three dimensions of the construct “corrupt intention.” As a result, 18 items were eliminated, yielding a final solution consisting of 15 items grouped into three factors (see Table 1). These factors accounted for 55% of the variance and demonstrated reliability indicators above 0.75 (ω total = 0.87 [0.86, 0.90]). Thus, the third version of the CIS was established.

**Table 1:** Factor loads in the third version of the CIS

Factors and items	PBC	ATB	SN	*h*^2^
Perceived behavioral control (PBC)				
35. I think I would ask a family member in a better position to get a job for a favor	.76	.09	-.10	.61
36. If I had a family member working in a hospital, I would ask them to help me to receive faster care	.91	-.08	.04	.79
37. If I had a relative working in the public sector, I would ask them to facilitate a procedure	.90	.01	.04	.86
38. If I had a contact in a hospital, I would ask for a bed or room for me or a sick family member	.87	-.11	.07	.72
39. If I held an important position in a public or private organization, I think I would employ my family and friends	.70	.19	-.09	.61
47. I would like to have contacts that would allow me to solve any problem related to public institutions	.56	.28	-.02	.55
Attitude toward behavior (ATB)				
3. I think that sometimes it is necessary to cheat	-.06	.63	.10	.40
5. To get what I want, I can do anything	.02	.62	.01	.40
7. I believe that I can do something illegal for my personal gain if no one is trureally harmed	.11	.59	.09	.45
11. I believe that he who does not cheat does not advance	-.10	.74	.01	.49
14. I think that every person has his price, what is necessary is to know what it is	.14	.67	-.10	.53
Subjective norms (SN)				
21. In our society, it is customary to give an improper bribe, gift, or payment	.04	-.06	.73	.54
22. It is normal in our society that something extra is paid to receive a better public service	-.04	0	.94	.88
31. To receive a better public service, you have to give something in return	.19	.32	.44	.51
32. One must pay something extra to receive a better service	.26	.29	.44	.55
Eigenvalues	3.92	2.46	1.88	
Proportion of variance	.26	.16	.13	
PBC (*M* = 17.1, *SD* = 5.7)	-			
ATB (*M* = 11.3, *SD* = 3.9)	.49	-		
SN (*M* = 12.4, *SD* = 3.8)	.35	.21	-	
*ω *[95% CI]	.90 [.88,.91]	.74 [.70,.78]	.87 [.86,.90]	

*N* = 369, *ω* Omega coefficient

## Study 2

### Methods

#### Participants

A total of 500 adult volunteers (62% women, 38% men) between the ages of 18 and 75 (*M* = 25.7, *SD* = 11.3) participated in the study (see Supplementary Material 4). The majority (85%) resided in Lima, while the remaining 15% lived in other regions. Most participants had a university education (75%). In terms of occupation, 67.4% were university students, 18% were salaried employees, 9.8% were self-employed, and 4.8% were unemployed at the time of the survey. The majority identified as mestizo (73%), and only 3.8% reported political party affiliation.

To determine the minimum required sample size a priori for this study and the next, we used Soper’s (2024) software for structural equation modeling. The calculation accounted for the number of observed and latent variables in the model, the anticipated effect size (0.20), the significance level (0.05), and the desired statistical power (1-β = 0.90). The analysis indicated that a total sample size of *N* = 382 would be required for the study, a threshold we were able to exceed.

#### Instruments

In addition to the sociodemographic data form used in Study 1, the third preliminary version of the CIS was used. This scale was made up of 15 items grouped into three factors: six items for *perceived behavioral control* (PBC), five items for the dimension *attitude toward behavior* (ATB), and four items for *subjective norms* (SN). The answer options remained the same as in the first version.

#### Procedure

In the fourth stage, we made changes to the test format, considering the reduction of items to confirm the factorial structure. The application was virtual (as in Study 1) and face to face. The latter was carried out in educational and work centers through the collaboration of professional psychologists who worked in business, educational, and health institutions. We eliminated 30 respondents because they were under 18 years of age, leaving the 500 volunteers analyzed. The same ethical aspects of Study 1 were followed.

#### Data analysis

A confirmatory factor analysis (CFA) using the robust maximum likelihood method was conducted to validate the internal structure of the CIS. Fit indices included χ^2^, CFI (Comparative Fit Index), TLI (Tucker‒Lewis Index), RMSEA (Root Mean Square Error of Approximation), and SRMR (Standardized Residual Mean Square), evaluated using Hu and Bentler’s (1999) criteria: good fit (CFI/TLI ≥ 0.95, RMSEA ≤ 0.05, SRMR ≤ 0.06) and adequate fit (CFI/TLI ≥ 0.90, RMSEA/SRMR ≤ 0.08). To select the most appropriate model among the factorial models compared, we considered both theoretical coherence with the proposed framework (TPB) and statistical criteria, including the chi-square difference test (Δχ^2^) and the Bayesian Information Criterion (BIC). A model with a greater increase in χ^2^ (for nested models) and a lower BIC value (Vrieze, 2012) would indicate a better-fitting model.

### Results

The highest response mean was for Item 21 *(“In our society, it is customary to give an improper bribe, gift, or payment”*; *M* = 3.5; *SD* = 1.2), while the lowest was for Item 11 (*“I believe that he who does not cheat does not advance”*; *M* = 1.86; *SD* = 1). The asymmetry values varied between -0.59 (Item 22, *“It is normal in our society that something extra is paid to receive a better public service”*) and 1.26 (Item 11), while the kurtosis values ranged between -1.18 (Item 32, *“One must pay a little extra to receive better service”*) and 1.12 (Item 11). Correlation coefficients between items ranged from 0.17 to 0.74 (see Table B of Supplementary Material 3).

Based on the results of Study 1, Model 1 (M1) was specified as a model composed of 15 items grouped into three correlated factors (PBC, ATB and SN). Model 2 (M2) was re-specified from Model 1 (M1) following a thorough analysis of the content of each item and the modification indices, which identified the most significant improvements to the model. As a result, items 32 and 35 were removed due to high error correlations with other elements in the model, while item 47 was discarded for loading on more than one factor. Model 3 (M3) was specified as a second-order model derived from M2, where the correlations among the three first-order factors are explained by the second-order factor: corrupt intent. Additionally, we proposed Model 4 (M4) as a bifactor model derived from M2, in which a general latent factor (corrupt intent) accounts for the primary variance in the items, while the specific factors (independent of each other) capture the shared variance among them.

M1 exhibited moderate fit indices: χ^2^ = 267.41 (*df* = 87, *p* < 0.001), CFI = 0.92, TLI = 0.90, RMSEA = 0.064 (90% CI [0.057, 0.072]), SRMR = 0.051, BIC = 21,471. M2 and M3 showed identical fit indices, as they are statistically equivalent and generate the same estimated covariance matrix: χ^2^ = 115.14 (*df* = 51, *p* < 0.001), CFI = 0.97, TLI = 0.96, RMSEA = 0.046 (90% CI [0.031, 0.060]), SRMR = 0.044, BIC = 17,289. Compared to M2 and M3, M4 exhibited slightly superior fit indices (χ^2^ = 86.54, *df* = 42, *p* < 0.001, CFI = 0.98, TLI = 0.97, RMSEA = 0.041, 90% CI [0.024, 0.057], SRMR = 0.030, BIC = 17,316). However, it also showed a significant reduction in χ^2^ compared to M2 and M3 (Δχ^2^ = 22.7; *p* = 0.006) alongside an increase in BIC relative to these models.

According to the Theory of Planned Behavior (TPB) and based on model comparison using fit indices such as Δχ^2^ (for models M2 and M4) and BIC (for all four models), as well as the results from additional fit indices (CFI, RMSEA, and SRMR), we concluded that the final version of the CIS consists of 12 items distributed across three correlated factors: perceived behavioral control, attitudes toward the behavior, and subjective norms (see Appendix).

Table 2 shows the factorial loadings of the items for the final version of the CIS, which ranged between 0.48 and 0.86. The magnitudes of the correlations were 0.41, 0.51 and 0.52 (*p* < 0.001). The three factors presented coefficients *ω* equal to or greater than 0.75.

**Table 2:** Factor loadings, interfactorial correlation, and internal consistency of the final version of the CIS

Factors and Items	PBC	ATB	SN
Perceived behavioral control (PBC)			
36. If I had a family member working in a hospital, I would ask them to help me to receive faster care	.86		
37. If I had a relative working in the public sector, I would ask them to facilitate a procedure	.86		
38. If I had a contact in a hospital, I would ask for a bed or room for me or a sick family member	.81		
39. If I held an important position in a public or private organization, I think I would employ my family and friends	.54		
Attitude toward behavior (ATB)			
3. I think that sometimes it is necessary to cheat		.68	
5. To get what I want, I can do anything		.59	
7. I believe that I can do something illegal for my personal gain if no one is truly harmed		.69	
11. I believe that he who does not cheat does not advance		.48	
14. I think that every person has his price, what is necessary is to know what it is		.60	
Subjective norms (SN)			
21. In our society, it is customary to give an improper bribe, gift, or payment			.68
22. It is normal in our society that something extra is paid to receive a better public service			.84
31. To receive a better public service, you have to give something in return			.65
PBC (*M* = 12.2, *SD* = 4)	-		
ATB (*M* = 11.3, *SD* = 4)	.53	-	-
SN (*M* = 9.9, *SD* = 3)	.51	.42	-
*ω *[95% CI]	.85	.74	.76
	[.82,.86]	[.70,.77]	[.71,.79]

*N* = 500, *ω* Omega coefficient

## Study 3

### Methods

#### Participants

Nonprobabilistic sampling was carried out. We also used the same software to calculate the sample size that we used in the second study. A total of 619 adults (63.3% women, 36.7% men) aged between 18 and 76 years (*M*_age_ = 25.2, *SD*_age_ = 10.9) participated (see Supplementary Material 5). A total of 87.9% reported residing in Lima, and 12.1% reported residing in other departments of Peru. The majority of participants had higher education at a university (78.5%). Over two-thirds (69.5%) were university students, 18% were dependent workers, 9.4% were independent workers, and 3.1% had no occupation at the time of the survey. The majority identified themselves as mestizo (71.6%), and only 4.5% said they belonged to a political group.

#### Instruments

##### Sociodemographic data form

The same data form from previous studies were used.

##### Corrupt Intention Scale

The final version of CIS consisted of 12 items grouped into three factors: perceived behavioral control (PBC, four items), attitude toward behavior (ATB, five items), and subjective norms (SN, three items). In this sample, adequate internal structure validity evidence was confirmed (*χ*^*2*^ = 181.65, *df* = 51, *p* < 0.001; CFI = 0.94, TLI = 0.92, RMSEA = 0.064 (90% CI [0.055]. 074]), SRMR = 0.059; *λ* between 0.44 and 0.88), and satisfactory reliability (*ω* = 0.85, 0.69, and 0.77 for PBC, ATB, and SN, respectively).

##### Dirty Dozen Dark Triad (DDDT; Jonason & Webster, 2010)

DDDT measures the dark triad of personality: Machiavellianism, Psychopathy, and Narcissism. The Peruvian version of Copez-Lonzoy et al. (2019) presents satisfactory evidence of validity and internal consistency. In our sample, we found adequate evidence of validity (*χ*^*2*^ = 76.2, *df* = 51, *p* < 0.001; CFI = 0.99, TLI = 0.99, RMSEA = 0.020 (90% CI [0.013, 0.041]), SRMR = 0.047; *λ* between 0.58 and 0.76) and satisfactory reliability indicators (*ω* = 0.82, 0.72, and 0.83 for Machiavellianism, psychopathy, and narcissism, respectively).

##### Dispositional Greed Scale (DGS, Seuntjens et al., 2015)

A seven-item scale was used to measure the dispositional tendency of people to be greedy. We used the version from Estrada-Mejía et al. (2023). In our third sample, there was adequate internal structure validity evidence (*χ*^*2*^ = 62.4, *df* = 27, *p* < 0.001; CFI = 0.99, TLI = 0.99, RMSEA = 0.046 (90% CI [0.031, 0.061]), SRMR = 0.050; *λ* between 0.61 and 0.79) and satisfactory reliability (*ω* = 0.88).

##### Dispositional Envy Scale (DES) (Smith et al., 1999)

The eight items of the DES measure a person’s disposition toward envy. We used the version from Mola et al. (2014). We found adequate evidence of internal structure validity (*χ*^*2*^ = 73, *df* = 20, *p* < 0.001; CFI = 0.97, TLI = 0.96, RMSEA = 0.065 (90% CI [0.050, 0.082]), SRMR = 0.086; *λ* between 0.62 and 0.74) and satisfactory reliability (*ω* = 0.86).

#### Procedure

In the fifth phase, we sought evidence of mean invariance and convergent validity. For this last aspect, we identified and chose the variables that were associated with corrupt behavior and tested them. Then, we developed a battery of tests for virtual and face-to-face applications. We followed the same recruitment procedures and ethical aspects as for Study 2. Forty-three responses were eliminated because they were answered by children under 18 years of age, leaving a database of 619 cases.

#### Data analysis

The same procedures, measures, and criteria used in Study 2 were used to evaluate the fit of the model for women and men. The measurement invariance test was performed with multigroup CFAs, checking a series of hierarchically nested tests: configurational invariance, metric invariance, scalar invariance, and strict invariance (Meredith, 1993). Changes in CFI less than 0.010 (Δ CFI < 0.010) and less than 0.015 in RMSEA (Δ RMSEA < 0.015) provide evidence of invariance between the least restricted and the most restricted models (Chen, 2007). In addition, Pearson’s correlation coefficient (r) was calculated to evaluate bivariate relationships between the CIS and the chosen variables. The effect size criteria described by Cohen (1988) were used for these correlations.

### Results

#### Measurement invariance

Table 3 shows adequate adjustment indices for the factorial structure of the CIS in women and men. Evidence of measurement invariance is also observed between the two groups, considering the sequence of invariance models compared with the reference model (configurational).

**Table 3:** Adjustment indices for the CIS and invariance models according to sex

CIS	*χ*^2^ (*df*)	CFI	TLI	RMSEA 90% CI	SRMR	*Δχ*^2^ (Δ*df*)	|ΔCFI|	|ΔRMSEA|
Women	153.95*(51)	.93	.91	.072 [.060, .084]	.067	-	-	-
Males	85.55* (51)	.95	.93	.055 [.036, .073]	.060	-	-	-
Configurational	236.15*(102)	.93	.92	.065 [.055, .075]	.060	-	-	-
Metric	244.73*(111)	.93	.92	.062 [.053, .072]	.065	8.01^+^(9)	0	.003
Scalar	262.80*(120)	.93	.92	.062 [.053, .071]	.066	17.78* (9)	.003	.001
Strict	281.12*(132)	.92	.92	.060 [.051, .069]	.068	20.10^+^ (12)	.005	.001

*N* = 619*, χ*^*2*^ = chi-squared, *df* degrees of freedom, *CFI* Comparative fit index, *TLI* Tucker‒Lewis index, *RMSEA* Root mean square error of approximation, *SRMR* Standardized root mean square residual, Δχ^2^ Differences in chi-squared, Δdf Differences in degrees of freedom, |ΔCFI| Absolute change in CFI, |ΔRMSEA| Absolute change in RMSEA

^+^*p* > .05; *p < .05

#### Evidence of validity based on the relationship with other variables

The total CIS score and all its dimension scores were positively correlated with all the variables studied (Table 4). These correlations had a medium effect size, except for the dispositional envy variable, for which a small effect size was found.

**Table 4:** Correlation of CIS score with the studied variables

Variable	Total (CIS) (*M* = 33.5;* SD* = 8.4)	PBC (*M* = 12.4;* SD* = 3.9)	ATB (*M* = 11.2;* SD* = 3.7)	SN (*M* = 9.8;* SD* = 3)
1. Machiavellianism (*M* = 7.6; *SD* = 3)	.45**	.31**	.50**	.23**
2. Psychopathy (*M* = 7.8; *SD* = 3.1)	.35**	.24**	.39**	.18**
3. Narcissism (*M* = 10; *SD* = 3.7)	.40**	.35**	.34**	.24**
4. Dispositional greed (*M* = 27.2; *SD* = 8.1)	.45**	.37**	.38**	.30**
5. Dispositional envy (*M* = 13.9; *SD* = 5.9)	.27**	.15**	.33**	.15**

*N* = 619, *PBC* Perceived behavioral control, *ATB* Attitude toward behavior, *SN* Subjective norms

^**^ p < .001

## General discussion

In the evaluation of some variables related to moral behavior, corrupt behavior is usually difficult to evaluate and measure directly, possibly because people tend to deliberately conceal such behavior. Therefore, the use of theoretical approaches and indirect measures, such as corrupt intent, can better detect indicators of corrupt behavior.

The present study reports a brief scale that was carefully developed to measure corrupt intent. The CIS is composed of 12 polytomous items, and we believe that it fills part of an instrumental gap in the exploration of corrupt behavior, a scourge that is present in all continents and countries of the world to a greater or lesser extent, generating wealth for a few and poverty for many human beings.

Although there are some instruments to measure corrupt behavior, these instruments have been created specifically for acts of corruption in business contexts (Leong & Lin, 2009), address knowledge about corruption (Tan et al., 2016), and measure moral development in the context of corruption (Guerrero-Martelo et al., 2018). Their theoretical approach is based on personality traits (Agbo & Iwundu, 2016), or they address attitudes toward corruption in descriptive terms (Orellana & Bossío, 2021). These perspectives differ from those of the present study. In addition, it should be noted that many of the tests used to measure corrupt behavior only have partially explained psychometric properties (Ponce-Díaz et al., 2024), an aspect that may limit the evaluation of the rigor of these instruments.

The results obtained indicate that the CIS has an internal structure comprising three dimensions, which are consistent with the Theory of Planned Behavior (TPB) proposed by Ajzen (1991), the theoretical framework adopted in this study. Consequently, the items that make up the test assess the three dimensions established by the model: perceived behavioral control, attitude toward the behavior, and subjective norms. Furthermore, evidence was found supporting the calculation of a total score, which represents the construct of corrupt intention. The empirical justification for this total score may be useful for interpreting results within the framework of evaluation processes or multivariate research studies.

According to the results, a higher score on attitude toward behavior indicates a higher degree of positive assessment of corrupt behavior (e.g., “I think that sometimes it is necessary to cheat”). Likewise, a higher score in the subjective norm dimension implies that the person presents a set of internalized normative beliefs that reflect the perceived pressure of the reference group (society) to participate in corrupt behaviors (e.g., “In our society, it is customary to give a bribe, gift or improper payment”). A high score on the PBC factor indicates a strong perception of one’s ability to engage in corrupt behaviors (e.g., “If I occupied an important position in a public or private organization, I think I would employ my family and friends”). According to Ajzen (2020), this perception is shaped by the set of behavioral control beliefs held by the individual. These beliefs encompass the influence of external factors, such as legal barriers, financial resources, or holding a high-ranking position in an institution, as well as internal factors, including knowledge, physical ability, and decision-making power. Altogether, high scores in these three dimensions suggest a greater degree of corrupt intent and therefore a greater probability of engaging in corrupt behavior.

The CIS presents evidence of configurational, metric, scalar, and strict invariance regarding the sex of the participants. These findings suggest that corrupt intention is expressed in concrete indicators and that its understanding does not depend on the sex of the participants since the same meanings are attributed to the items and both women and men use the same conceptual framework of the item to issue their response. Therefore, if in future studies the application of the CIS produces differences between men and women, it is likely that this difference will be attributed to the real difference in corrupt intention and not to the bias of the instrument. These results can be interpreted in the framework of studies on sex and corrupt behavior (e.g., Bauhr & Charron, 2021; Waylen & Southern, 2021).

The CIS presents evidence of validity, given its relationship with other variables, since positive correlations were found with machiavellianism, psychopathy, narcissism, dispositional greed, and dispositional envy. Previous studies reported relationships between corrupt behavior and some of these variables (Gu et al., 2021; Li et al., 2023; Nugraha & Etikariena, 2021; Zhao et al., 2016). Although other techniques or instruments have been used in studies to measure corrupt behavior, our findings are consistent with the evidence available on the subject and suggest the complex nature of corrupt behavior and its study. The interactions of corrupt behavior with other psychological factors reported in our study and in previous studies can be considered for a better understanding of the phenomenon and interventions against it.

Limitations of this study include the characterization of the samples. Among the three groups of participants, there was a preponderance of young participants, with fewer middle-aged adults or older adults. This situation limited the analysis of measurement invariance with respect to the age group. Similarly, there was limited variety in the place of residence or occupation of the participant, so the possible influence of these variables on the factorial structure of the CIS could not be reported. We hope that future studies will confirm the psychometric properties of the scale and verify its measurement invariance with the indicated variables. It is important to note that this study was based on Classical Test Theory. Future research should apply Item Response Theory to assess the explanatory power of each item.

An additional limitation of this study is that the discriminant validity of the Corrupt Intent Scale (CIS) was not assessed in relation to other instruments measuring dishonest behaviors. Therefore, it is crucial for future research to include this evaluation to determine whether the items of the CIS, as well as the scale as a whole, specifically capture corrupt intent or if they overlap with other types of dishonest behaviors.

Despite the aforementioned limitations, we believe that the CIS offers certain advantages over other psychological measures that assess corrupt behavior, corrupt intention, or related constructs. First, its development is based on a theoretical framework that explains behavior through the construct of intention, which has substantial empirical support (Hagger et al., 2022; Lidong et al., 2025; Somoray et al., 2024). Second, its items capture attitudinal, normative, and behavioral control beliefs regarding an individual’s willingness to engage in corrupt acts, without explicitly using the term corruption or its derivatives, thereby minimizing social desirability bias. Third, the CIS was developed following methodological recommendations outlined in specialized guidelines (American Educational Research Association et al., 2014; Muñiz & Fonseca-Pedrero, 2019), and its psychometric validity was assessed using various statistical methods across three independent samples. Finally, the inclusion of measurement invariance analysis for sex is a key feature for comparative studies, an aspect that has not been addressed in previous instruments.

One of the key contributions of the CIS to the literature on corruption measurement lies in its ability to address the psychological dimension underlying the decision to engage in corrupt acts: intention. This scale allows for the assessment of attitudes, subjective norms, and perceived control—key factors in the formation of corrupt intent—thus providing a more robust predictive framework (Ajzen, 1991). Moreover, the CIS could bridge macro- and micro-level analytical approaches by identifying psychological patterns before intentions materialize into actions, thereby enriching research on ethics, integrity, and the theory of planned behavior.

The CIS could also be used in experimental research, assessing how different interventions, such as ethics or decision-making programs, impact the reduction of corrupt intentions before they translate into actual behavior. In addition, both public and private organizations could use this tool in their selection and training processes, in conjunction with other measures, to ensure that employees are not only evaluated in terms of technical competencies, but also in terms of their ethical propensity, which could contribute to more transparent and accountable work environments.

In the public policy field, having the CIS and similar instruments could better inform the design of preventive policies by enabling early identification of individuals or groups at risk of engaging in corruption. This could translate into the implementation of more effective educational and training programs aimed at strengthening personal integrity and reducing social acceptance of corruption.

## Conclusions

In conclusion, we created the CIS, an instrument to measure corrupt intent. Its construction process was based on TPB, a theory that has shown theoretical and empirical consistency. The CIS is the product of a systematic process of analysis and shows evidence of adequate validity and reliability that supports its use to indirectly measure corrupt behavior. We believe that the validation of a scale to measure corrupt intent is a key advance in organizational psychology, social psychology and business ethics, offering an evidence-based tool to assess predispositions towards corrupt behaviors. This type of instrument allows the identification of psychological factors that contribute to these intentions, facilitating more effective preventive interventions. In addition, its application can contribute to the development of public policies and corporate strategies focused on promoting ethical and sustainable organizational cultures.

## Supplementary Information

Supplementary Material 1.

Supplementary Material 2.

Supplementary Material 3.

Supplementary Material 4.

Supplementary Material 5.

## Data Availability

The datasets generated and/or analysed during the current study are available in the Mendeley Data repository, 10.17632/53bfjf6snt.1.

## Appendix

### Final version of the Corrupt Intention Scale (CIS)

**Instructions:** Below you will find some statements that describe aspects of human behavior. Please respond to each of them indicating your agreement or disagreement, taking into account the extent to which they describe you. To do this, use the following scale:

1: Strongly disagree, 2: Disagree, 3: Undecided, 4: Agree, 5: Strongly agree

**Table:**

**Items ***	**1**	**2**	**3**	**4**	**5**
1. I think that sometimes it is necessary to cheat. (3) (*Pienso que a veces es necesario hacer trampa*)					
2. To get what I want, I can do whatever I want. (5) (*Para obtener lo que quiero, puedo hacer cualquier cosa*)					
3. I believe that I can do something illegal for my personal gain if no one is truly harmed. (7) (*Creo que puedo hacer algo ilícito para mi beneficio personal si nadie sale realmente perjudicado*)					
4. I believe that he who does not cheat does not advance. (11) (*Creo que el que no hace trampa, no avanza*)					
5. I think that every person has his price, what is necessary is to know what it is. (14) (*Pienso que toda persona tiene su precio, lo que hace falta es saber cuál es*)					
6. In our society, it is customary to give an improper bribe, gift, or payment. (21) (*En nuestra sociedad, es una costumbre dar una coima, regalo o pago indebido*)					
7. It is normal in our society that something extra is paid to receive a better public service. (22) (*Es normal en nuestra sociedad que se pague algo extra para recibir un mejor servicio público*)					
8. To receive a better public service, you have to give something in return. (31) (*Para recibir un mejor servicio público, hay que dar algo a cambio*)					
9. If I had a family member working in a hospital, I would ask them to help me receive faster care. (36) (*Si tuviera un familiar trabajando en un hospital, le pediría que me ayude para recibir una atención más rápida*)					
10. If I had a relative working in the public sector, I would ask them to facilitate a procedure. (37) (*Si tuviera un familiar trabajando en el sector público, le pediría que me facilite un trámite*)					
11. If I had a contact in a hospital, I would ask for a bed or room for me or a sick family member. (38) (*Si tuviera un contacto en un hospital, le pediría una cama o cuarto para mí o algún familiar enfermo*)					
12. If I held an important position in a public or private organization, I think I would employ my family and friends. (39) (*Si ocupara algún cargo importante en una organización pública o privada, creo que daría empleo a mis familiares y amigos*)					

* All items are scored directly. The original numbering of each item appears in parentheses

## Abbreviations

CIS: Corrupt Intention Scale
TI: Transparency International
IBCS: Intercultural Business Corruptibility Scale
CAS: Corruption Awareness Scale
CPS: Corruption Propensity Scale
DIT-SF: Defining Issues Test
SATC: Scale of Attitudes Toward Corruption
TPB: Theory of Planned Behavior
ATB: Attitude Toward the Behavior
SN: Subjective Norm
PBC: Perceived Behavioral Control
SDGs: Sustainable Development Goals
EFA: Exploratory Factor Analysis
CFA: Confirmatory Factor Analysis
CFI: Comparative Fit Index
TLI: Tucker‒Lewis Index
RMSEA: Root Mean Square Error of Approximation
SRMR: Standardized Residual Mean Square
DDDT: Dirty Dozen Dark Triad
DGS: Dispositional Greed Scale
DES: Dispositional Envy Scale
