# Supplementary material for: Psychological insight into corruption: construction and validation of the Corrupt Intention Scale (CIS)
Source: Psicol Reflex Crit. 2025 May 20;38:15. doi: 10.1186/s41155-025-00352-3 (PMC12092907; doi:10.1186/s41155-025-00352-3)
Supplement: Supplementary file 2 — Supplementary Material 2. [file 41155_2025_352_MOESM2_ESM.pdf]

Supplementary material 2
Analysis codes in the R program

1. Database creation (Data 1 = 369)

```
rm(list = ls())
datosbase<-read.spss("Data1.sav", to.data.frame=T,use.value.labels=FALSE)
myvars<-c('C1','C2','C3','C4','C5','C6','C7','C8','C9','C10',
        'C11','C12','C13','C14','C15','C16','C17','C18','C19',
        'C20','C21','C22','C23','C24','C25', 'C26','C27','C28',
        'C29','C30','C31','C32','C33','C34', 'C35','C36','C37',
        'C38','C39','C40','C41','C42','C43', 'C44','C45','C46','C47')
datosfa=datosbase
datosfa<-datosbase[myvars]
```

2. Descriptive analysis (Data 1 = 369)

```
describe(datosfa)
head(Data1,n=3)
```

3. Creation of a database for exploratory factor analysis (Data 1 = 369)

```
rm(list = ls())
datosbase<-read.spss("Data1.sav", to.data.frame=T,use.value.labels=FALSE)
myvars<-c('C1','C3','C4','C5','C6','C7','C10',
        'C11','C12','C13','C14','C21','C22','C23','C24',
        'C29', 'C31','C32','C33','C34', 'C35','C36','C37',
        'C38','C39','C40','C41','C42','C43', 'C44','C45','C46','C47')
datosfa=datosbase
datosfa<-datosbase[myvars]
```

4. Multivariate normality test (Data 1 = 369)

```
result <- mvn(data=datosfa, mvnTest = "mardia")
result
mvn(data = datosfa, mvnTest = "mardia")
```

5. Exploratory factor analysis (EFA) (Data 1 = 369)

```
KMO(datosfa)
cortest.bartlett(datosfa)

parallel.m<-fa.parallel(datosfa,fm='minres',fa='fa')
efa<-fa(nfactors=3,cor='poly',fm="minres",rotate="oblimin",datosfa)
print(efa$loadings,cutoff=0.40)
print(efa)
fa.diagram(efa)
```

6. Reliability (Data 1 = 369)

```
omega(datosfa, nfactors = 3, plot = F)
ci.reliability(data=datosfa, type = "omega", interval.type = "perc",
           B = 100)
```

7. Confirmatory factor analysis (CFA) (Data 2 = 500)

```
model1 <-  " F1  =~ C35+C36+C37+C38+C39+C47
           F2  =~ C3+C5+C7+C11+C14
           F3  =~ C21+C22+C31+C32"
model2 <-  " F1  =~ C36+C37+C38+C39
           F2  =~ C3+C5+C7+C11+C14
           F3  =~ C21+C22+C31 "
Model3<-  " F1  =~ C36+C37+C38+C39
           F2  =~ C3+C5+C7+C11+C14
           F3  =~ C21+C22+C31
           General =~ F1+F2+F3"
sem.fit = sem(model3,estimator="MLR",data=datosbase)
summary(sem.fit,fit.measures=T,standardized=T)
semPaths(sem.fit,whatLabels="std",layout="tree",edge.label.cex=0.9,rotation=2,nCharNodes=15,
       sizeLat=7,sizeMan=7,style="lisrel")
head(modificationindices(sem.fit)[order((modificationindices(sem.fit))$mi,decreasing=TRUE),],20)
```

8. Reliability for the final version (Data 2 = 500)

```
reliability(sem.fit)
```

9. Measurement Invariance (Data 3= 619)

```
#Ajuste para grupo, previo a MI
Data3G1=subset(datosfa,SEXO==1)
sem.fit = sem(model3,estimator="MLR",data=base4G1)
summary (sem.fit, fit.measures=T, standardized=T)
Mydata=read.spss("Analisis Factorial.sav",to.data.frame=T,use.value.labels=F)
Mydata=subset(Mydata,DeEstudio==1)
Data34G2=subset(datosfa,SEXO==2)
sem.fit = sem(model3,estimator="MLR",data=base4G2)
summary (sem.fit, fit.measures=T, standardized=T)
#Haciendo MI con MLR
sem.fit.Config<-cfa(model3,data=datosfa,estimator="MLR",group="SEXO")
sem.fit.Weak..<-
cfa(model3,data=datosfa,estimator="MLR",group="SEXO",group.equal=c("loadings"))
sem.fit.Strong<-
cfa(model3,data=datosfa,estimator="MLR",group="SEXO",group.equal=c("loadings","intercepts"))
sem.fit.Strict<-
cfa(model3,data=datosfa,estimator="MLR",group="SEXO",group.equal=c("loadings","intercepts","residuals"))
summary (sem.fit.Config, fit.measures=T, standardized=T)
summary (sem.fit.Weak.., fit.measures=T, standardized=T)
summary (sem.fit.Strong, fit.measures=T, standardized=T)
summary (sem.fit.Strict, fit.measures=T, standardized=T)
a=compareFit(sem.fit.Config,sem.fit.Weak..)
b=compareFit(sem.fit.Weak..,sem.fit.Strong)
c=compareFit(sem.fit.Strong,sem.fit.Strict)
summary(a)
```

```
summary(b)
summary(c)
```

10. Evidence of validity based on relationship with other variables (Data 3 = 619)

```
round(cor(datosfa),2)
```
