# Supplementary material for: Psychological insight into corruption: construction and validation of the Corrupt Intention Scale (CIS)
Source: Psicol Reflex Crit. 2025 May 20;38:15. doi: 10.1186/s41155-025-00352-3 (PMC12092907; doi:10.1186/s41155-025-00352-3)
Supplement: Supplementary file 3 — Supplementary Material 3. [file 41155_2025_352_MOESM3_ESM.pdf]

Supplementary material 3

Correlation coefficients of the items

Tabla A

Correlation coefficients of the items of the second version of the CIS

Variable	C1	C3	C4	C5	C6	C7	C10	C11	C12	C13	C14	C21	C22	C23	C24	C29	C31	C32	C33	C34	C35	C36	C37	C38	C39	C40	C41	C42	C43	C44	C45	C46	C47
1. C1	—																																
2. C3	.09	—																															
3. C4	.24	.24	—																														
4. C5	.32	.37	.30	—																													
5. C6	.17	.21	.30	.22	—																												
6. C7	.17	.39	.22	.32	.49	—																											
7. C10	.20	.16	.25	.22	.49	.41	—																										
8. C11	.15	.37	.19	.36	.16	.38	.25	—																									
9. C12	.25	.24	.23	.28	.36	.34	.37	.27	—																								
10. C13	.23	.11	.21	.23	.25	.30	.35	.22	.38	—																							
11. C14	.24	.32	.21	.39	.32	.43	.32	.42	.30	.29	—																						
12. C21	.14	.15	.33	.11	.27	.18	.25	.08	.26	.19	.05	—																					
13. C22	.11	.22	.23	.11	.26	.24	.21	.11	.25	.24	.10	.66	—																				
14. C23	.25	.23	.27	.25	.31	.32	.32	.21	.25	.17	.30	.29	.37	—																			
15. C24	.30	.18	.22	.33	.25	.35	.31	.18	.28	.26	.33	.24	.24	.30	—																		
16. C29	.22	.24	.28	.33	.34	.32	.40	.30	.31	.29	.35	.30	.30	.36	.41	—																	
17. C31	.28	.24	.22	.29	.30	.30	.30	.29	.33	.23	.32	.32	.44	.32	.36	.44	—																
18. C32	.20	.26	.22	.30	.28	.29	.30	.25	.35	.25	.34	.33	.45	.37	.35	.42	.74	—															
19. C33	.15	.31	.14	.23	.27	.37	.30	.32	.30	.20	.44	.13	.18	.35	.35	.39	.44	.52	—														
20. C34	.13	.35	.11	.35	.21	.32	.19	.37	.18	.13	.42	.07	.16	.40	.37	.36	.39	.45	.66	—													
21. C35	.19	.24	.17	.26	.25	.32	.25	.18	.20	.23	.33	.14	.12	.34	.37	.32	.32	.38	.43	.48	—												
22. C36	.25	.20	.22	.24	.33	.33	.32	.19	.29	.32	.30	.24	.27	.38	.35	.36	.38	.41	.39	.42	.62	—											
23. C37	.28	.26	.19	.27	.31	.34	.34	.23	.27	.27	.36	.25	.29	.42	.43	.37	.43	.48	.46	.48	.65	.80	—										
24. C38	.26	.20	.18	.21	.31	.27	.29	.17	.29	.27	.30	.30	.26	.35	.38	.37	.35	.38	.38	.39	.54	.77	.73	—									
25. C39	.20	.21	.13	.28	.24	.38	.22	.26	.18	.18	.37	.15	.18	.29	.43	.33	.34	.37	.44	.51	.59	.55	.62	.59	—								
26. C40	.10	.39	.14	.29	.29	.44	.28	.41	.28	.20	.47	.07	.17	.36	.40	.40	.32	.35	.57	.56	.38	.36	.46	.32	.54	—							
27. C41	.18	.33	.21	.33	.35	.41	.32	.36	.30	.26	.39	.25	.37	.35	.34	.39	.57	.65	.51	.51	.42	.47	.52	.43	.48	.50	—						
28. C42	.20	.25	.19	.27	.32	.41	.31	.27	.24	.22	.37	.19	.24	.36	.42	.39	.40	.46	.49	.52	.62	.63	.73	.61	.73	.54	.62	—					
29. C43	.11	.36	.19	.26	.29	.37	.29	.32	.33	.14	.39	.18	.27	.35	.33	.31	.44	.54	.48	.51	.43	.52	.54	.50	.51	.48	.61	.61	—				
30. C44	.18	.35	.21	.29	.33	.45	.30	.31	.30	.19	.40	.16	.27	.33	.37	.39	.35	.42	.56	.51	.36	.48	.49	.50	.48	.54	.57	.54	.62	—			
31. C45	.17	.24	.19	.23	.31	.24	.22	.22	.23	.11	.28	.10	.17	.28	.28	.31	.27	.28	.38	.35	.22	.22	.28	.29	.29	.41	.40	.36	.35	.38	—		
32. C46	.17	.23	.15	.28	.30	.26	.24	.27	.21	.10	.32	.12	.16	.26	.25	.30	.23	.28	.40	.40	.32	.32	.31	.29	.36	.35	.34	.36	.34	.38	.37	—	
33. C47	.28	.26	.22	.37	.34	.37	.31	.25	.31	.27	.44	.17	.17	.34	.39	.34	.38	.40	.47	.44	.50	.50	.59	.48	.55	.47	.48	.62	.47	.47	.35	.45	—

N = 369

Tabla B

Correlation coefficients of the items of the third version of the CIS

Variable	C3	C5	C7	C11	C14	C21	C22	C31	C32	C35	C36	C37	C38	C39	C47
1. C3	—														
2. C5	.42	—													
3. C7	.48	.41	—												
4. C11	.35	.27	.32	—											
5. C14	.38	.34	.42	.30	—										
6. C21	.21	.20	.21	.18	.19	—									
7. C22	.19	.18	.21	.09	.27	.59	—								
8. C31	.21	.19	.25	.23	.28	.40	.54	—							
9. C32	.29	.27	.22	.21	.33	.34	.47	.67	—						
10. C35	.26	.25	.30	.16	.27	.27	.32	.29	.32	—					
11. C36	.29	.20	.28	.25	.31	.28	.35	.34	.34	.60	—				
12. C37	.32	.29	.31	.18	.30	.30	.36	.31	.33	.62	.74	—			
13. C38	.31	.19	.30	.16	.27	.28	.35	.36	.36	.57	.70	.69	—		
14. C39	.24	.30	.30	.28	.27	.17	.20	.26	.20	.56	.46	.47	.42	—	
15. C47	.33	.39	.40	.23	.38	.28	.33	.36	.39	.53	.52	.54	.52	.47	—

N = 500
